# Analysis of Data Interaction Process Based on Data Mining and Neural Network Topology Visualization

**DOI:** 10.1155/2022/1817628

**Published:** 2022-06-29

**Authors:** Nina Dai

**Affiliations:** Shanghai Donghai Vocational & Technical College, Shanghai 200241, China

## Abstract

This paper addresses data mining and neural network model construction and analysis to design a data interaction process model based on data mining and topology visualization. This paper performs preprocessing data operations such as data filtering and cleaning of the collected data. A typical multichannel convolutional neural network (MCNN) in deep learning techniques is applied to alert students' academic performance. In addition, the network topology of the CNN is optimized to improve the performance of the model. The CNN has many hyperparameters that need to be tuned to construct an optimal model that can effectively interact with the data. In this paper, we propose a method to visualize the network topology within unstable regions to address the current problem of lacking an effective way to layout the network topology into specified areas. The technique transforms the network topology layout problem within the unstable region into a circular topology diffusion problem within a convex polygon, ensuring a clear, logical topology connection, and dramatically reducing the gaps in the area, making the layout more uniform beautiful. This paper constructs a real-time data interaction model based on JSON format and database triggers using message queues for reliable delivery. A platform-based real-time data interaction solution is designed by combining the timer method with the original key. The solution designed in this paper considers the real-time accuracy, security and reliability of data interaction. It satisfies the platform's initial and newly discovered requirements for data interaction.

## 1. Introduction

As the scale and technology of information systems continue to improve and develop, the degree of informatization is increasing, the construction of information technology is vigorously promoted, the number of information technology applications running in each enterprise is increasing, and the data exchange between the applications is becoming more and more frequent. However, due to the different periods of application systems in the enterprise or the use of various technologies and other reasons, the data sources in each application system have become an “information island” that cannot directly interact [1]. The research of data interaction is to provide solutions for sharing and system integration of various data and information in the enterprise, to provide a global data view, global data authority view, and perfect data exchange service for the application systems in the enterprise, to solve the problem of “information silo” in many application systems in the enterprise, and to enable various data resources to interoperability among application systems [2]. In response to the emerging needs in the platform and the limitations of previous research, a JSON and message queue-based multisource heterogeneous real-time data interaction model are constructed based on the core of the industry chain system platform by using the characteristics of JSON format data with less redundancy and combining message queue technology and database trigger design. The model meets the requirements of real-time data interaction, realizes flexible configuration of data interaction, and ensures the integrity, accuracy, and security of data interaction.

Estimation of network topology has a long history; in the earliest days of network creation, due to the low complexity and small network size, the standard means of estimation was the internal node collaboration-based measurement method based on protocol standards for probing. However, with the development of network technology, internal node collaboration-based network topology estimation methods are no longer applicable: on the one hand, the information obtained through these methods of measurement is often incomplete as the network size increases; on the other hand, some of the network protocols on which these methods rely have been discontinued as people become more aware of network security [3]. To reduce the reliance on internal nodes, people have applied laminar imaging techniques to network topology estimation, opening a new field of network laminar imaging. The most mature practice in this approach is the network laminar imaging technique for smooth networks. In this method, researchers assume that the individual parameters of the network are stable, and it is relatively easy to perform network topology estimation under such a precondition [4]. However, in the natural network environment, the network is nonstationary, and the individual parameters of the network are constantly changing. The network topology information measured by this method often has a significant error from the actual one, so the practical application of this method is not very meaningful.

Convolutional neural networks have been widely used in image recognition, segmentation, speech recognition, etc. [5]. One of the main reasons deep learning has attracted increasing attention in recent years is that convolutional neural networks have made incredible progress in the IMAGE NET race, with a 10% improvement in inaccuracy. But with the development of deep learning, a wide variety of convolutional neural network models have been proposed, and neural networks based on new algorithms, loss functions, and topologies flood the field: AlEXNet, GOOGLE NET, LENET, DROPOUT, etc. [6]. But in these developments, the application of new techniques is based on the same topology under the same topology, with a hodge-podge of similarities; we guess that if we analogize the training method of human brain information transfer and fundamentally change the network structure, the neural network constructed by this idea should have a better training performance and be able to be used in the field of deep learning [7]. The neural network built by this idea should have better training performance and brain-like information transfer characteristics to a certain extent.

## 2. Related Works

Network topology visualization technology has been flourishing with the Internet, and there have been many diverse and in-depth research results and application techniques. And with the development of emerging technologies such as graph computing and VR, researchers in various fields have made significant progress in visualization, which still has a relatively broad development prospect [8]. CAIDA is one of the most well-known research institutions that have made significant research progress in Internet topology visualization. CAIDA, or the Global Collaboration for Internet Data Research, is a famous and productive research institution in Internet topology research [9]. CAIDA describes itself as developing new visualization techniques to display Internet data transmission networks to understand the Internet better. CAIDA has made some progress on many Internets topology-related research topics, especially the work on autonomous domain-level topology and topology visualization, which is also covered later in this paper [10]. In addition to the research work, this organization has developed and published many practical visualization tools, enriching the relevant agencies in network topology visualization, which is very useful. For example, CAIDA has released the Walrus tool, a tool that enables interactive visualization of large-scale directed graphs in 3D space, ideal for visualizing tree-like graphs with less than 100,000 nodes, based on the principle of abstracting topological graphs into spanning trees and embedding the spanning trees in a sphere containing the Euclidean projection of a 3D hyperbolic space for rendering. In addition to visualization tools, CAIDA also has many visualization research results, proposing to embed the Internet in a two-dimensional Hilbert space according to routing information to implement routing policies within the Internet instead of routing tables to solve the current problem of routing table inflation within the Internet, and also providing different strategies for discovering association structures in complex networks [11]. CAIDA has gradually focused on network topology measurement and topology analysis in recent years. The most important recent visualization work is the Internet Autonomous Domain Core Distribution Map, which plots each autonomous domain in polar coordinates on a circle; the closer to the center of the process, the more autonomous domain clients there are, and the closer to the periphery of the ring the fewer independent domain clients there are, showing the importance of each autonomous domain in this intuitive way.

The origin of data mining dates to the 20th century, when database technology was already more mature [12]. Still, people's needs were rising, and simple database queries and other operations could no longer meet people's needs, so under the constant impetus of increasing demand, it gradually developed into a merger of database technology and traditional artificial intelligence technology, that is, by using standard database technology to the database technology is used to manage and store a large amount of data, which we call database management system [13]. Artificial Intelligence (AI) technology is used to automate the analysis and processing of large amounts of data and information and uncover the fundamental knowledge behind a large amount of data. A comprehensive discipline called knowledge discovery in information technology, and resource management systems have been created [14]. The obvious is to use advanced data mining and technical means to collect and discover all data-based information processes in an informational database that stores a large amount of data, so data mining plays a crucial role in this discipline [15]. Gradually, data mining has developed a set of mining models covering association, classification, clustering, etc., and is already a very mature technical and theoretical framework for discovering more about the knowledge behind the data.

Compared with traditional data interaction technology, real-time data interaction technology is proposed later, and there are relatively few studies on real-time data interaction technology [16]. No mature solutions have been formed; only some practice and exploration on the design of real-time data interaction based on traditional data interaction technology have been conducted [17]. Scholars such as Wang et al. proposed a mechanism for scheduling data updates based on factors such as record M Quinn Francis studied the real-time data interaction in the Sonic Air system [18]. Shi et al. use Java programming technology to design the process of real-time data exchange in a socket way based on XML [19]. Hazarikaet al. propose an agent-based model for distributed real-time data exchange, which provides a detailed study of data communication, task scheduling, transaction control, and data access in distributed real-time data exchange [20]. The mechanisms of data communication, task scheduling, transaction control, data access, etc., in distributed real-time data exchange are discussed in detail [21]. Xin et al. studied the data change capture method and proposed a log-based change data capture framework [22]. At the same time, the software industry has also offered a related patent that uses the enterprise service bus ESB combined with Web Service technology to orchestrate and integrate service components to achieve real-time data interaction [23]. The patent proposes a method and system for realizing real-time data exchange interface, which makes the interaction between multiple systems complete real-time interface functions through a unified protocol without the need to reformulate the interface between systems, greatly simplifying the implementation of the interface.

## 3. Design of Data Interaction Process Model Construction Based on Data Mining and Neural Network Topology Visualization

### 3.1. Data Mining and Neural Network Optimization Model Construction

The core of a convolutional neural network is the convolutional and pooling layers, and most of the computation is done in these two layers. Several optimizations for these two layers are analyzed here first [24]. The convolutional filter of the primary convolutional neural network is a generalized linear model of local image blocks, which can be used well for abstraction when the instances of the underlying concepts are linearly separable.

#### 3.1.1. Tiled Convolution

The weight sharing mechanism in convolutional neural networks can significantly reduce the number of network parameters, but it may also limit the model to learning to train other kinds of invariants. Tiled convolutional neural networks are a variant of convolutional neural networks that tile blocks of images and multiple features map to learn rotation and to scale-invariant features. Separate convolutional kernels are known in the same layer and can implicitly learn complex invariants by performing square root pooling on adjacent units.

#### 3.1.2. Extended Convolution

Extended convolutional neural networks, relative to traditional convolutional neural networks, add a hyperparameter to the convolutional layer. By inserting zeros between the filters, extended convolutional neural networks can increase the size of their receptive fields and allow the network to cover more relevant information. This critical feature is used in tasks requiring a large receptive field when making predictions. Formally, a one-dimensional extended convolutional neural network with expansion *l* convolves a signal *F* with a kernel and kernel sizer, as shown in the following equation:

(1) $$\begin{array}{c} F_{k-l}=\sum_{r=1}\frac{\sqrt{k_{r}-k}}{F_{t-l}+1}. \end{array}$$

#### 3.1.3. Network in Net

**Figure 1:**
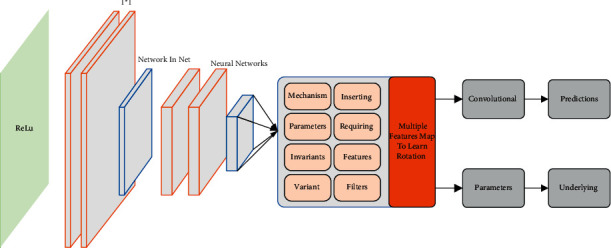
Multilayer perceptron convolutional layer and linear convolutional layer.

The most crucial feature of Network in Net (NIN) compared to traditional convolutional neural networks is that it does not have a linear filter in the convolutional layer but uses a small network instead, such as a multilayer perceptron convolutional layer, which enables it to approximate a more abstract representation of the underlying concept. The overall structure of NIN is a superposition of such small networks. The general form of NIN is a superposition of such small networks, as shown in Figure 1, which shows the difference between multilayer perceptron convolutional layers and linear convolutional layers. The feature map of the linear convolutional layer is computed as

(2) $$\begin{array}{c} z_{ijk}=\int \frac{w_{k}-x_{ij}}{b_{k}-o}, \end{array}$$

where *z*_*ijk*_ is the activation value of the kth feature map at the point (*I*, *J*), *X*_*i, j*_ is the input pixel block centered at the end (*I*, *J*), and *W*_*k*_ and *B*_*k*_ are the weight vector and bias term of the kth filter. The feature map of the convolutional layer of the multilayer perceptron compared to this is computed as

(3) $$\begin{array}{c} Z_{ijk}^{n}=\sum_{k=1}\frac{w_{k}^{t}}{b_{k}+o}, \end{array}$$

where *n* is the number of layers of the multilayer perceptron convolutional layer and *A*_*ij*_ is equivalent to *X*_*i*, *j*_ in the multilayer perceptron convolutional layer, a 1^*∗*^1 convolutional kernel is added after the conventional convolutional layer, equal to the cross-channel parameter pooling operation of ReLu. Therefore, the multilayer perceptron convolutional layer can also be regarded as a cascaded cross-channel parameter pooling of the traditional convolutional layer.

Pooling is an essential concept in convolutional neural networks, which reduces the computational burden by reducing the number of connections between convolutional layers.

(1) Lp pooling: Lp pooling is a biologically inspired process modeled on complex cells. It provides a better generalization concerning maximal pooling, formulated as follows:
  (4) $$\begin{array}{c} y_{\left(i-j\right)}=\sum_{R_{ij}}\left(a_{m}-nk\right). \end{array}$$
(2) Mixed pooling: mixed pooling is a mixture of average pooling and maximum pooling, given by the following equation:
  (5) $$\begin{array}{c} y_{ijk}=\sum \left[\lambda \max\left(a_{mnk}\right)+\sum \frac{1}{\sqrt{R_{ij}-1}}\right]. \end{array}$$
A random value of 0 or 1 corresponds to using average or maximum pooling, whose value is recorded in the forward propagation and used in the backward propagation operation. Hybrid pooling solves the overfitting problem better and outperforms both average and maximum pooling.
(3) Random pooling: Random pooling is a pooling method based on Dropout and Drop Connect. Random pooling randomly selects activation values based on a multinomial distribution, unlike average pooling, which takes the average value in each pooling region, unlike maximum pooling, which takes the total value in each pooling region. Its multinomial distribution ensures that the feature map's average and maximum activation values are likely to be selected. Stochastic pooling first calculates the probability *p* for each region *R*_*j*_ by normalizing the activity within the area.
  (6) $$\begin{array}{c} P=\sum_{k-r_{j}}\frac{a}{a_{k}}. \end{array}$$

After obtaining the probability distribution, a multinomial distribution based on *p* is sampled, a location *l* is selected within the region, and the pooling activation is set *Y*_*j*_=*A*_*l*_. Compared to maximum pooling, random pooling avoids overfitting due to random components.

**Figure 2:**
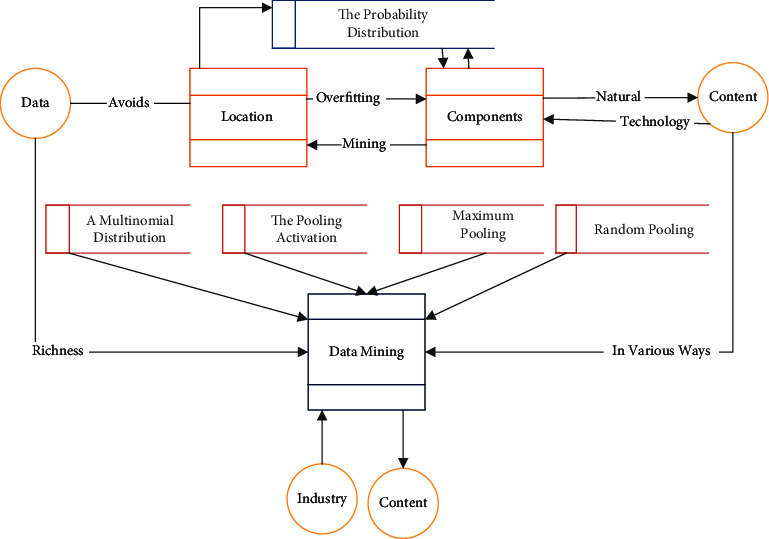
The whole process of data mining.

The extensive research and widespread use of data mining can be seen as a result of the natural evolution of information technology. As a multidisciplinary field, data mining can be defined in various ways. Even the definition of the term “data mining” itself does not fully cover the richness of its content. Strictly speaking, “data mining” in the industry is a broad concept, which should be more accurately called “knowledge mining from data” or “knowledge discovery in data,”; while “data mining” in the narrow sense of “data mining” is only an essential step in the whole process of knowledge discovery. The entire process of data mining is shown in Figure 2.

1. Data cleansing: The raw data used for knowledge discovery in real applications are usually incomplete and, except for special applications such as outlier analysis, noise should be eliminated, inconsistent data removed, anomalous and erroneous values corrected, and uncertain or incomplete values completed.
2. Data integration: combining data from multiple sources and in different forms into one.
3. Data selection: extraction and analysis of task-related data from the database.
4. Data transformation: transform and unify the data into a form suitable for mining through aggregation operations.
5. Data mining: as an essential step, the most central operation in the whole process, using automatic and intelligent methods to extract data patterns
6. Pattern assessment: screening of exciting patterns representing knowledge based on a specific measure of interest.
7. Knowledge representation: using visualization and knowledge representation techniques to show the mined knowledge to users.

Data cleansing, integration, selection, and transformation are collectively referred to as data preprocessing and used as the construction process for data warehousing. It is worth noting that data warehousing is not a mandatory operation. Sometimes, data transformation and unification are performed before data selection, especially in the case of data warehousing. Data normalization may also be required to obtain a more miniature representation of the original data without sacrificing its integrity.

The architecture of fast Text is different from most popular large neural networks with a complex hierarchical structure. Quick Text only contains three layers: input, implicit, and output [25]. The input layer is used as the upper part of the model, and the n-gram word vector is obtained by superimposing all the words of the Text and then averaged to generate a vector to characterize the Text. The idea behind the superimposed word vectors is the traditional bag-of-words method CBOW, which treats the Text as a collection of words. The remaining part is the lower part of the model, which is used as a hierarchical classifier for the SoftMax multiclassification task. The input of this classifier is the output of the upper part of the model, i.e., the vector characterizing the Text. Therefore, the critical idea of fast Text is to obtain a text vector after implementing the superposition averaging operation on the n-gram vector and then use this vector to complete the fast multiclassification task; the expressions are as follows:

(7) $$\begin{array}{c} b_{k-1}=\sum \frac{\sqrt{b_{k-n}}-1}{\sqrt{b_{k}-k}}. \end{array}$$

For the text classification task in data mining, a more interpretable model NNF is designed by combining the base model fast Text. Analogous to a random forest, each network in this model has the same architecture, such as CNN and LSTM. LSTM is Memory, so the main feature of LSTM is to have some memory capacity, and hence most of the time, it is used to deal with sequences, such as processing a sentence or a video. CNN is mainly strong in dealing with a single picture, and the association between the front and back is not so strong in a sequence, but of course, 3D CNN can sometimes be used to deal with video. For the classification problem, the output of each network in the forest is a single neuron and represents a particular class. There are as many networks in the woods as there are classification categories. The corresponding category label is set as a positive category for each network in the forest. All other category labels are placed as harmful categories. Each wood network is trained using the categorized text data in a loop. During the prediction period, the highest prediction weight is obtained for one and only one network, i.e., the predicted Text belongs to that category and not to the category to which the other networks belong. This model focuses on the structural interpretability of neural networks compared to other mega networks like VGG, Image Net, etc. Traditional neural network: suppose we use a fully connected layer with 128 units, then we need 300 × 300 × 128 = 11520000 parameters. Convolutional neural network: assume we use a 5 × 5 × 3 filter; for different regions, we share the same filter, so we share the same set of parameters; one filter has 75 parameters; suppose we use ten filters, then we need 750 parameters.

### 3.2. Data Interaction Model Design for Data Mining and Neural Network Topology Visualization

**Figure 3:**
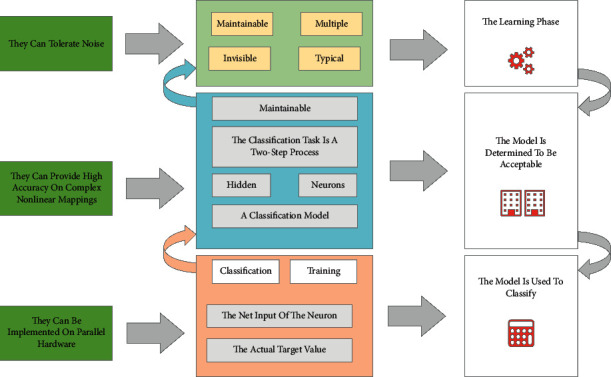
Complex data types for data mining.

In data mining, neural networks are often used as an advanced method for data classification. Using neural networks for data analysis has the following advantages: (1) they can tolerate noise; (2) they can provide high accuracy on complex nonlinear mappings; (3) they can be implemented on parallel hardware; and (4) they are highly maintainable, can be easily updated with new data, and can be easily automated. The general approach to the classification task is a two-step process. First, a classification model is built based on existing data, called the learning phase. Then, the accuracy of the model is determined to be acceptable, and if so, the model is used to classify the new data, called the classification phase. The complex data types for data mining are shown in Figure 3.

**Figure 4:**
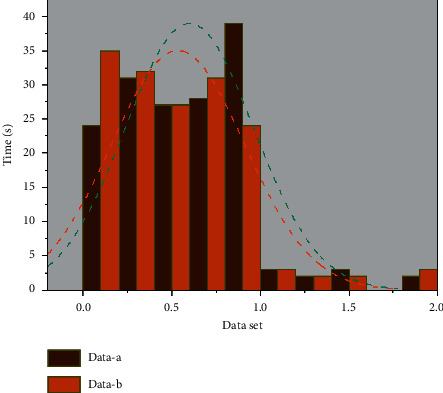
Comparison of neural network optimization model data.

Before training the neural network, each neuron's weights and bias values in the constructed network need to be initialized first. The next step is forward propagation, where the training tuples are provided to the network's input layer, and the inputs through the input units do not change. Each neuron's input and output values are computed in the hidden and output layers, giving the typical form of neurons in the invisible or production layers. In fact, in addition to the input layer neurons, each neuron usually has multiple inputs *X*_*k*_, and these input values are the outputs of the neurons of the previous layer that connect it. Where *V*_*k*_ is called the net input of the neuron, the result *Y*_*K*_ is obtained through the activation function [26]. There are many types of nonlinear activation functions, and the output of forwarding propagation often deviates from the actual target value, requiring the use of backward propagation algorithms, which continuously update and adjust the weights and bias values of the neurons in the network layer by layer, starting from the error between the output and the target value. Backward propagation uses the gradient descent method to search for the set of weights. These weights fit the training data such that the error between the prediction of the sample and the known target value of the tuple is minimized, where 2 is the learning rate, usually taking a constant value of [0, 2]. The learning rate can help avoid getting trapped in local minima in the decision space, thus helping to find the optimal global solution. Convolutional neural network optimization model data comparison is shown in Figure 4.

In this visualization system, to make the business logic clear and the data flow clear, we borrowed the layered form of the familiar MVC pattern to realize the system construction. The core of MVC is to separate front-end display, business logic, and data into three layers, each of which is independent of the other and provides interfaces to the outside world to facilitate communication between the layers. This modular approach decouples the system from the complex data flow, makes it easy for developers to sort out the data flow and changes, and effectively reduces the complexity of the code and logic. According to the MVC layering idea, this system can be divided into page visualization, business logic, and data layers. The visualization layer seen by users corresponds to the V layer in MVC, which is directly user-oriented and operated by users. When the user interacts with the visualization graphics, the corresponding business logic layer will judge and respond to the user's operation [27]. If the user adds, changes, or checks the data; the business logic layer detects that the data has changed and notifies the visualization layer to re-render and respond to the user's operation. Suppose the user's process does not affect the data. In that case, the logic layer will not notify the visualization layer to render, significantly improving the system performance and reducing the browser's rendering of the page. In this visualization system, the implementation of visualization mainly relies on GO.JS and charts. They are open-source libraries, and they can both render data in JSON format to achieve visual graph drawing.

**Figure 5:**
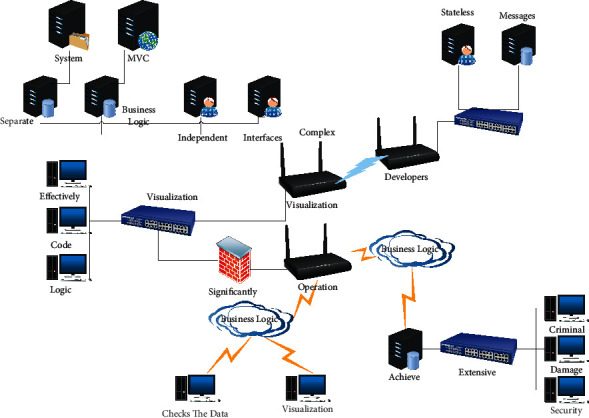
Interactive data flow.

Since accurate business data will be exposed to the extensive network environment of the Internet in the process of data interaction, they may be attacked by criminal elements. These attacks may lead to business data leakage, tampered business data, server backdoor vulnerability, or even direct damage to the server. Hence, the security of these business data becomes an issue that must be considered in this paper [28]. First, consider the user legitimacy of the interaction messages. Since the interaction message is sent using the HTTP protocol, a stateless and encryption-free flat text transmission protocol, any network node can directly receive and read out the content of the interaction message and forge it. Therefore, it is designed to verify that the sender's identity is a legitimate user of the platform and that the sender user is the user to which the published data interaction configuration belongs. The flow of the interaction data is shown in Figure 5.

The interaction message publisher obtains the information saved during platform login in the local configuration and requests the Token for authentication from the platform-side server and caches it locally. The authentication Token is attached to the interaction message and sent to the message subscriber. After the interaction message subscriber gets the authentication Token, it authenticates the Token to the platform-side server and caches it locally. After multiple uses, the Token is removed from the store, and a new Token is requested from the platform server.

## 4. Analysis of Results

### 4.1. Data Interaction Model Testing for Network Topology Visualization

**Figure 6:**
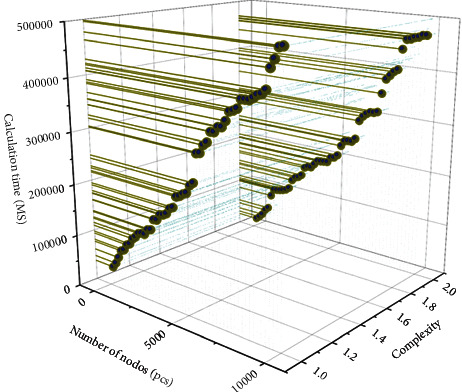
Comparison of computational complexity.

The system function test mainly detects whether each functional module of the network topology visualization system can operate normally. The network topology visualization system is based on browser server architecture with visualization as the core [29]. It can verify whether the system functions appropriately through the interface display. The parallel force-oriented layout algorithm improves the problem of the high computational complexity of the basic force-oriented network topology layout algorithm by multithreaded design and optimized annealing algorithm. In the experiment, 7 threads are set for parallel computation according to the number of CPU cores in the experimental host. The maximum number of iterations of 50 is introduced as the parameter of the annealing function in the annealing algorithm. The topology layout of a different number of nodes is tested. To exclude the interference caused by the additional complexity of other networks, we simplify the connection of edges, use a more straightforward graph structure in the test process, and specify the degree of all nodes in the test data as 2. The test results are shown in Figure 6.

When the number of network topology nodes is below 500, there is no significant difference in the running time of the two algorithms. In the test below 20 nodes, there is even a situation where the layout time of the parallel force-oriented algorithm is higher than the layout time of the force-oriented algorithm, mainly because the creation and switching of multiple threads in a parallel force-oriented algorithm will cause the loss of resources. Still, from 1000 nodes, the advantage of a similar algorithm is reflected. However, from 1000 nodes onwards, the edge of the parallel algorithm comes out. A more significant difference in the layout time of the two algorithms appears. The more the number of nodes and the more complex the topology, the more the parallel layout algorithm has the advantage.

**Figure 7:**
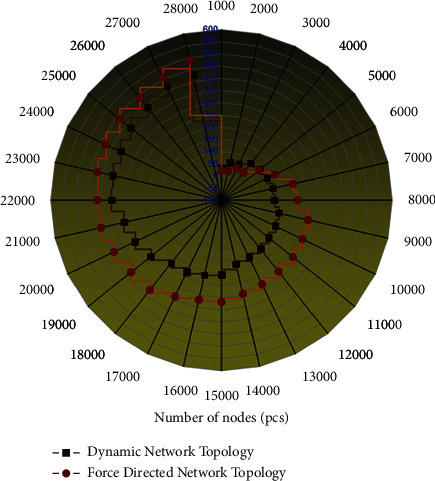
Performance comparison between network topology re-layout algorithm and force-oriented layout algorithm.

The dynamic network topology layout algorithm is mainly applied to the satellite-based time-varying network in the integrated network between heaven and earth. For the changing network topology, how to maintain the cognitive continuity of the snapshot change of the network topology by the holding user and accelerate the layout speed to meet the real-time requirements. In this experiment, the comparison of the layout time consumption of the satellite dynamic network topology re-layout algorithm and the layout time consumption of the force-oriented network topology algorithm is carried out. For effective comparison, a topology graph with an initial topology of 500 nodes is selected for illustration in this paper. At the same time, to reduce the influence of other factors, the degree of nodes in the initial topology graph is specified as 2. The addition and deletion of edges and nodes are carried out continuously several times, the average value is calculated, and the network topology is re-layout. A comparison of the algorithm's performance and the force-oriented layout algorithm is shown in Figure 7.

After data processing, the association rules are then analyzed using the FP-growth data mining algorithm. First, we need to set the minimum support and confidence in advance. Because the values of minimum support and minimum trust are tried continuously during the experiment, it is not appropriate to give the correct values at the beginning, and it is necessary to try several times and analyze the results of each investigation to finally determine the importance of minimum support and minimum confidence that meet this experiment. First, we started from a relatively small value and tried to make the minimum support equal to 0.5, while the minimum confidence percentage was 0.8. After analysis, we got hundreds of association rules, and the amount of association data obtained was too small. There were many meaningless rules, and it was difficult to analyze them, so we needed to adjust the minimum support value again. After continuous attempts, we finally determined that the minimum support was set to 1.0. After analyzing the results, we can see that each security level has a common point in the assessment items with association rules, so we can know that the mining results of association rules are reasonable and meaningful. On the other hand, the data in the database is not comprehensive, and more data will be added one after another.

### 4.2. Data Interaction Model Implementation for Network Topology Visualization

**Figure 8:**
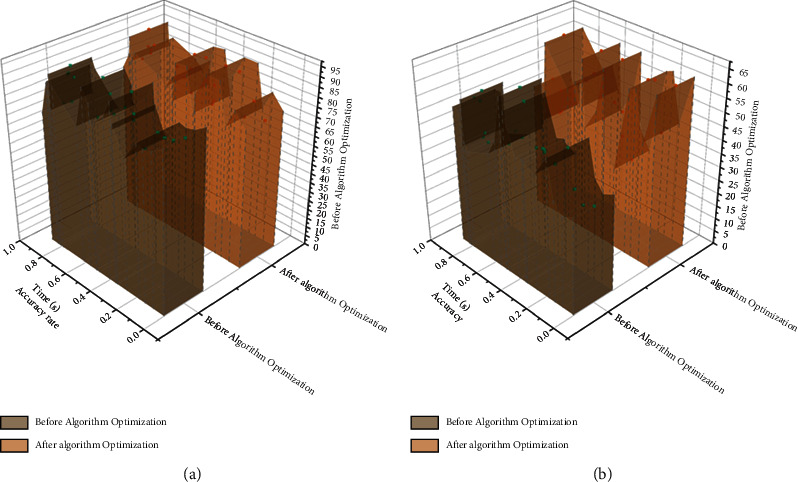
Comparison of the optimized work procedures of the client-side of the data interaction process module.

The Data Interaction Process module implementation is a form program launched to perform initial runtime checks and start each type of data monitor, inherited from the pure virtual base class Define Proc and managed by Define Proc Manager. Changeset Helper assists the data monitor in extracting and confirming the changed data. When the data monitor detects the change, it is sent to the corresponding Worker for preprocessing before sending, formatted as Manager type, and delivered to the Message Queue module for reliable delivery. Other classes provide auxiliary operations, logging, and interface displays to operate the data interaction [30]. After receiving the interaction message, the data interaction subscriber performs unpacking and reverse processing and finally applies it to the local database while rolling back the operation if an error occurs and returning an error message to trigger retransmission. In using changes to the local database, the system decides how to apply the changes to the database according to the number of changes. When all the changes occur in one data table and the number is large, Bulk Copy is used for batch application to improve the efficiency of database operation; if the changes occur in scattered data tables and the number is small, a single update operation is used to avoid the time consumed by Bulk Copy to parse the structure of the changed data table. Comparison chart of the client work's procedures of the data interaction process module after optimization is shown in Figure 8.

**Figure 9:**
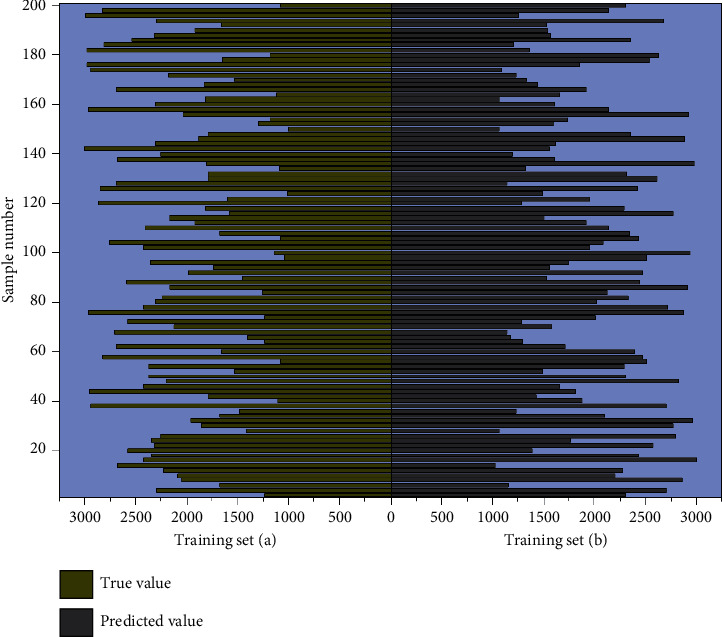
Comparison of real-time data interaction components interacting with real-time data.

The results of several models show that the overall prediction accuracy of the PCA-SVM model tends to increase as the number of principal components increases. However, the original purpose of using this method is to reduce the dimensionality of the indicators, so even though the accuracy of the PCA-SVM prediction model can reach 0.81377 when the number of principal components is 15, the extraction of 15 main features from 17 indicators at this time only reduces two dimensions, and this process disrupts all the original indicators. The efficiency of dimensionality reduction is too low currently. The number of principal components extracted by the two principles of cumulative variance contribution rate greater than 84% and corresponding eigenvalue greater than 1 is 10 and 6, respectively. Although the purpose of dimensionality reduction is achieved to a certain extent, the prediction accuracy of the corresponding test set of single-party cost indicators is 0.43158 and 0.2136, respectively, which cannot meet the accuracy requirements in actual engineering. The real-time data interaction component interacts with real-time data pairs, as shown in Figure 9.

The management module is integrated into the industry chain collaboration platform as a configurable function module, divided into four parts: platform security authentication interface, platform system settings, business configuration management, and data interaction statistics display. Among them, the platform security authentication interface has no interface display. It is responsible for generating authentication tokens and verifying the authenticity of authentication tokens, which is realized by generating tokens through hashing user ID and time, saving them in the platform database, and comparing them with the identity tokens requested for verification to return the verification results. The platform system setting includes the database connection setting and global parameter setting of the platform, and the interface takes the database connection setting of the platform as an example. Data interaction statistics display provides a graphical display window for administrators to view the operation of real-time data interaction components, including logs of system operation, statistical collection of operation parameters, and real-time data view of interaction. The entity relationships of related My SQL tables are organized, and the data in these tables that are unstructured are classified. At the same time, the indexes and structured data information are stored in My SQL tables. Transfer the data that needs to be transferred to intermediate processes or tables, delete the My SQL-related data with the help of atomicity of transactions, and make backup nodes to ensure that the whole business will not be affected.

## 5. Conclusion

The visualization of network topology is becoming increasingly important with the successful development of the Internet. It has a wide range of application prospects in network topology analysis, security situational awareness, management, and Internet modeling. This paper takes the topological Internet space as the topological input. It uses its autonomous domain-level topology as the entry point to complete the research work on various visualization methods. A crucial possible direction is to apply not only advanced algebra, analytics, and geometry to solve the puzzle of neural network interpretability through theoretical analysis; data mining is a multidisciplinary discipline, and the use and combination of related algorithms and techniques vary depending on different application scenarios and needs. Whether it is possible to create universal data mining algorithms and application models is a beautiful topic, and if a breakthrough is achieved, it will also have a profound impact on the improvement of socioeconomic benefits; meanwhile, considering the business that does not require real-time data interaction in real-time, designing timer-type data interaction models; this paper combines the frontier domain interpretability of deep neural networks and constructs a structurally interpretable. This paper combines the frontier field interpretability of deep neural networks, creates NNF with interpretable structure model, and analyzes the reason of model structure interpretable. Through the experimental comparison with the base model fast Text, it is found that NNF has good performance and has a specific application value. In this paper, we design a hybrid storage model based on My SQL and Mongo DB, in which we briefly describe how to extract the data in it the specific data analysis and optimize the overall storage model by storing Mongo DB indexes by My SQL, etc., to reduce the overall service response time and improve the throughput of externally processed data. The extraction-type data interaction model is designed considering the requirement of full data synchronization. The secure and reliable delivery is achieved by using encryption and redundant checksum technologies to ensure the security and reliability of data interaction.

Data mining is a multidisciplinary discipline, and the use and combination of related algorithms and techniques vary depending on different application scenarios and needs. Whether it is possible to create universal data mining algorithms and application models is a beautiful topic, and if a breakthrough is achieved, it will also have a profound impact on the improvement of social and economic benefits; the application of the interpretability of neural networks in data mining will make an interesting research direction, and theoretical research will eventually serve specific needs and applications, and how to thoroughly combine the two in applications needs to be explored by scholars in the future. How to thoroughly mix the two in the application needs to be explored more deeply in the future.

## Data Availability

The data used to support the findings of this study are included within the article.
